# Supplementary material for: Sleep Spindle-Related EEG Connectivity in Children with Attention-Deficit/Hyperactivity Disorder: An Exploratory Study
Source: Entropy (Basel). 2023 Aug 22;25(9):1244. doi: 10.3390/e25091244 (PMC10530036; doi:10.3390/e25091244)
Supplement: Supplementary file 1 [file entropy-25-01244-s001.zip › suplementary_material_miladinovic.pdf]

Supplementary Materials:

1. Multivariate Autoregressive Models

A multivariate autoregressive model (MVAR) is a discrete-time, linear, and time-tnvariant model described by differential equations. The etymology of the name of these models is related to their capacity in predicting the future value of more input by means of linear regression.

Let y_n be a vector of stationary, in a broad sense, and ergodic stochastic processes with dimension $m \times N$, with N the number of samples of each process and m the number of processes. An MVAR of order p, $MVAR(p)$ is defined as

$$y(n) = -\sum_{K=1}^{p} A(k)y(n-k) + u(n) \tag{S1}$$

where $y(n) = [y_1(n), y_2(n), \ldots, y_m(n)]^T$ is the n-th sample of the fourth time series and p is the model order that represents the number of previous samples needed to describe the present sample of the process. $A(k)$ is a matrix ($m \times m$) containing the model coefficients related to the k-th lag. Therefore, A is a tridimensional matrix of $m \times m \times p$. Finally, $u(n) = [u_1(n), u_2(n), \ldots, u_m(n)]^T$, representing the n-th samples of white Gaussian noise with zero mean and covariance matrix Σ.

$$A(1) = \begin{bmatrix} a_{11} & a_{12} & a_{13} & a_{1m} \\ a_{21} & a_{22} & a_{23} & a_{2m} \\ \vdots & \vdots & \ddots & a_{3m} \\ a_{m1} & a_{m2} & a_{m3} & a_{mm} \end{bmatrix} \cdots A(p) = \begin{bmatrix} a_{11} & a_{12} & a_{13} & a_{1m} \\ a_{21} & a_{22} & a_{23} & a_{2m} \\ \vdots & \vdots & \ddots & a_{3m} \\ a_{m1} & a_{m2} & a_{m3} & a_{mm} \end{bmatrix} \tag{S2}$$

$$\Sigma = \begin{bmatrix} \sigma_{11}^2 & 0 & 0 & 0 \\ 0 & \sigma_{22}^2 & 0 & 0 \\ 0 & 0 & \ddots & 0 \\ 0 & 0 & 0 & \sigma_{mm}^2 \end{bmatrix} \tag{S3}$$

Equation (S1) could be rewritten in matricial form by using (S2) and (S3):

$$Y = A \cdot X + U \tag{S4}$$

where the U matrix of the inputs has a dimension of $m \times m$ and A has a dimension of $m \times m \times p$. Therefore the parameters of the model that have to be estimated are those of A and the four variances of Σ the matrix.

At n-th time instant, each time series is described as the weighted linear sum of the $n-1, \ldots, n-p$ previous values of all inputs, where the weights are the a_{ij} coefficients of (S2). The diagonal coefficients ($i = j$) represent the influence of the past values of the node on the future values of itself and are called self-loops. In contrast, the other matrix elements represent the cross-influences among the nodes. This means that the a_{ij} coefficient is the weight of the process j on the evolution of the process i, therefore $a_{ij} \neq a_{ji}$. These MVAR properties are fundamental for the evaluation of cortical connectivity.

If p is well defined, the identification of the model means evaluating the coefficients of A and of the covariance Σ matrices. By using Equation (S1), the prediction error of the model can be defined as the difference between the real value $y(n)$ and the predicted one $\widetilde{y}(n)$:

$$e(n) = y(n) - \widetilde{y}(n) = y(n) + \sum_{k=1}^{p} A(k)y(n-k) \tag{S5}$$

The optimum parameters for the model are those that minimize the variance in the prediction error; therefore, by applying the least square error method, the results are obtained by minimizing the cost function:

$$J(n,\Theta) = E\left[e^2(n)\right] \quad \text{with } \Theta \text{ parameters vector} \tag{S6}$$

The minimum of J is the point at which the partial derivatives of the unknown parameters are zero.

$$\frac{\partial J(\Theta)}{\partial A(k)} = 0 \quad \text{with } k = 1,\ldots,p \tag{S7}$$

Moreover, because the error prediction vector $e(n)$ can be assumed as an estimation of the input noise $u(n)$, the covariance matrix Σ can be calculated as the covariance matrix of the prediction error (that corresponds with the minimum of the cost function defined in (S6). Finally, the system consists of $p+1$ Yule–Walker equations, in $p+1$ unknowns matrices (p matrices for the a_{ij} coefficients and one for the covariance values of $u(n)$).

$$\begin{bmatrix} R(0) & R(1) & R(2) & \cdots & R(p) \\ R(1) & R(0) & R(1) & \cdots & R(p-1) \\ \vdots & \vdots & \vdots & \ddots & \vdots \\ R(p) & R(p-1) & R(p-2) & \cdots & R(0) \end{bmatrix} \begin{bmatrix} A(0) \\ \vdots \\ \vdots \\ A(p) \end{bmatrix} = - \begin{bmatrix} \Sigma \\ 0 \\ \vdots \\ 0 \end{bmatrix} \tag{S8}$$

where $A(0) = I$ is the identity matrix of dimension $p \times p$ and $A_{ij}(k) = -Aji(k)$. The $R(k) = E[y(n)y(n-k)]$ matrices are the auto-correlation matrices (dimension $p \times p$) of the $y(n)$ processes with the elements on diagonals representing the correlation of the m processes and others representing the cross-correlations. The estimation of the system parameters in (S8) could be assessed through the application of recursive algorithms, for example, the Levinson–Durbin.

1.1. Frequency domain MVAR

The application of the MVAR model allows information to be obtained in both time and frequency domains. Rearranging Equation (S1) and applying the Z-transform is the first step in obtaining the following frequency domain equation:

$$y(n) + \sum_{k=1}^{p} A(k)y(n-k) = u(n) \tag{S9}$$

$$Y(z) + \sum_{k=1}^{p} A(k)Y(z)z^{-k} = U(z) \quad \text{so} \quad Y(z)(I + A(z)) = U(z) \tag{S10}$$

From (S10), the transfer function of the model in z-domain is extracted:

$$H(z) = \frac{Y(z)}{U(z)} = \frac{1}{I + A(z)} = \frac{1}{\overline{A}(z)} \tag{S11}$$

$$substituting\ z = e^{j2\pi fTk}:$$

$$H(f) = \frac{Y(f)}{U(f)} = \frac{1}{\overline{A}(f)} \tag{S12}$$

where $\overline{A} = I + A$, with $\overline{A}(0) = I$ and $\overline{A}(k) = -A(k)$ for $k = 1,\ldots,p$, and where $H(f)$ and $\overline{A}(f)$ are matrices of dimension $p \times p$. $A(f)$ represents the Fourier Transform of the coefficients defined in (S12)

$$A_{ij} = -\sum_{k=0}^{p} \bar{a}_{ij}(k)e^{j2\pi fTk} \tag{S13}$$

The $H_{ij}(f)$ element represents the transfer function between the $i-th$ input and the $j-th$ output of the MVAR model at the frequency f with $H_{ij}(f) \neq H_{ji}(f)$, whereas the $A_{ij}(f)$ element is the transfer function between the $i-th$ input and $j-th$ output of the MVAR linear predictor at the frequency f with $A_{ij}(f) \neq A_{ji}(f)$.

The time series spectrum described by the MVAR model is given by

$$S(f) = |H(f)|^2\Sigma \tag{S14}$$

with $S(f)$ a matrix of dimension $p \times p$ that contains the spectral description of the time series of y. The spectral matrix is Hermitian, which means it has real components on the diagonal and conjugated complex values elsewhere, and is symmetric ($S_{ij}(f) = S_{ji}(f)$).

$$S(f) = \begin{bmatrix} S_{11}(f) & S_{12}(f) & \cdots & S_{1m}(f) \\ S_{21}(f) & S_{22}(f) & \cdots & S_{2m}(f) \\ \vdots & \vdots & \ddots & \vdots \\ S_{31}(f) & S_{32}(f) & \cdots & S_{mm}(f) \end{bmatrix} \tag{S15}$$

The elements on the main diagonal are the auto-spectrum of the processes $y_1, y_2, \ldots, y_m$, whereas the elsewhere elements are the cross-spectrum.

1.2. Optimum p order

The choice of the optimum order for a model is evaluated by using parsimony criteria (e.g. Schwarz–Bayes Criterion, Akaike Information Criterion, Hannan–Quinn Criterion) and then validated by analyzing the whiteness of the prediction error. The difference among the criteria is the severity used to penalize the order increment, thus varying the performance depending on the considered dataset. The most used is the Schwarz–Bayes Criterion, but it is applied in cases with a great number of samples; meanwhile, the Akaike Information Criterion has also been applied for small sample numbers. The equation of Akaike is described as follows:

$$AIC(p) = \ln(det(\Sigma)) + \frac{2}{N}pm^2 \tag{S16}$$

with N the number of samples, m the number of processes, and the first term is the logarithm of the determinant of the estimated noise covariance matrix. The order selection should be performed carefully, because a too-small model order can impair the frequency resolution; on the contrary, a too-large one can overfit the model. For the analysis of the EEG data, it has been demonstrated that a potentially optimal model was $p = 10$ [72,73], although some differences were identified for order in the range of 9 to 13.

2. Generalized Partial Direct Coherence

In 2001, Baccalà and Sameshima [38] proposed a novel approach to evaluate the Granger causality in the frequency domain based on MVAR models, which they named Partial Direct Coherence (PDC). They started from the concept of Direct Coherence (DC), which is a measure of how two structures are functionally connected, providing both the *"feedforward"* and *"feedback"* aspects of the relationship. DC is defined as

$$C_{ij}(f) = \gamma_i^H(f)\gamma_j(f) \tag{S17}$$

with $\gamma(f) \triangleq [\gamma_{i1}(f), \ldots, \gamma_{im}(f)]^T$, and H stands for Hermitian matrix.
In contrast with DC, PDC allows many time series to be analyzed simultaneously, thanks to the application of the MVAR, which provides all the direct structural information.

The PDC definition is based on the *partial coherence function*, $|K_{ij}(f)|^2$, that quantifies the relationship between pairs of signals, $x_i(n)$ and $x_j(n)$, when the influence due to all other $N-2$ time series is discontinued.

$$k_{ij}(f) = \frac{\overline{a}_i^H(f)\Sigma^{-1}\overline{a}_j(f)}{\sqrt{(\overline{a}_i^H(f)\Sigma{-}1\overline{a}_i(f))(\overline{a}_j^H(f)\Sigma{-}1\overline{a}_j(f))}} \tag{S18}$$

where Σ is the prediction error covariance matrix and $\overline{a}_k(f)$ is the $k-th$ column of the matrix $\overline{A}(f) = I - A(f) = [\overline{a}_1(f), \overline{a}_2(f), \ldots, \overline{a}_m(f)]$. From the analogy between (S17) and (S18), the general definition of the PDC factor from j to i is given by

$$\pi_{ij}(f) \triangleq \frac{\overline{A}_{ij}(f)}{\sqrt{\overline{a}_j^H\Sigma^{-1}\overline{a}_j(f)}} \tag{S19}$$

where $\overline{A}_{ij}(f)$ is the $i,j-th$ element of $\overline{A}(f)$. The PDCF depends mostly on the a_{ij} coefficients because

$$\overline{A}_{ij}(f) = \begin{cases} 1 - \sum_{r=1}^{p} a_{ij}(r)e^{-i2\pi fr} & \text{, if } i = j; \\ -\sum_{r=1}^{p} a_{ij}(r)e^{-i2\pi fr} & \text{otherwise} \end{cases} \tag{S20}$$

To remove the instantaneous Granger causality effects, (S19) can be rewritten as

$$\pi_{ij}(f) \triangleq \frac{\overline{A}_{ij}(f)}{\sqrt{\overline{a}_j^H\overline{a}_j(f)}} \tag{S21}$$

depending only on $a_{ij}(r)$ coefficients. Here, (S21) is the mathematical definition of *Partial Direct Coherence*(PDC) from j to i and holds the following normalization properties:

$$0 \leq |\overline{\pi}_{ij}(f)|^2 \leq 1 \qquad \text{and} \qquad \sum_{i=1}^{N} |\overline{\pi}_{ij}(f)|^2 = 1 \tag{S22}$$

for all $1 \leq j \leq N$.

The coefficient $\overline{\pi}_{ij}(f)$ delineates the coupling strength from a source structure j to structure i compared to all interactions of j with other structures; therefore, PDC classifies the interaction strength based on a given signal source. In the case of $i = j$, the coefficient $\overline{\pi}_{jj}(f)$ represents how much of the past signal $x_i(n)$ influences its present state. If the dynamic range of a time series is modified by gains, the PDC will not be able to correctly evaluate the direction of the information flow. To remove this problem, Baccalà and colleagues [40] defined a new Partial Direct Coherence estimator called Generalized Partial Direct Coherence (gPDC):

$$\pi_{ij}^{(u)}(f) = \frac{\frac{1}{\sigma_i}\overline{A}_{ij}(f)}{\sqrt{\sum_{k=1}^{N} \frac{1}{\sigma_k^2}\overline{A}_{kj}(f)\overline{A}_{kj}^*(f)}} \tag{S23}$$

where σ_i^2 refers to the variances in the error processes (S1). The new definition preserves the normalizations (S22).

The gPDC is invariant to scale and reduces variability for short time series. In addition, it is an instance of variance stabilization of the Granger causality and is better used in the analysis of models with large prediction errors or large disparities in the power spectra between multivariate channels [74].

3. MVAR application

Each piece of EEG, obtained using the method described in the pre-processing section, was aggregated by epoch type for each subject. This allows three 3D matrices, (*Pre*, *Dur*, and *Post*), to be accomplished for each subject with the dimension of $M \times T \times N$, where M is 19 (channels), T is 512 (sample points equal to 1 second EEG), and N varies between 6 and 10 (number of spindles of each subject). Hence, 51 matrices were obtained (3 matrices for each subject). The matrices were directly used to evaluate the MVAR model, since signals could not be stationary, and the Adaptive MVAR of SIFT Matlab toolbox was applied.

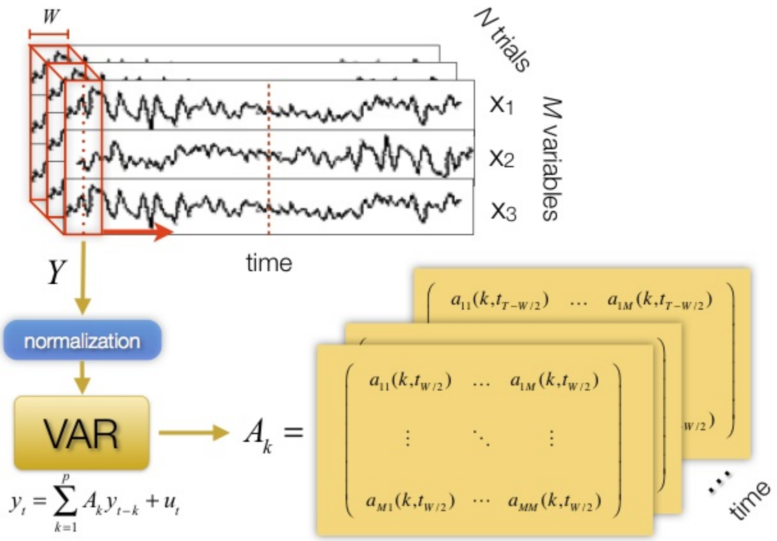

Figure S1. Explanation of the process for the calculation of the MVAR coefficients. The illustration on the top represents the EEG raw data of M variable, T time points, and N trials. W is the length of the sliding window that starts at the first time point of the signal, and steps until the end by a number of time points defined by the user.

AMVAR is a modification of the standard MVAR models that adds the possibility of using a sliding window over time and the number of spindles as trials of the same process (Fig. S1). Before starting the AMVAR analysis, the length and step of the sliding window and the order (p) of the model had to be chosen and a normalization of the signal had to be performed. Instead of arbitrarily defining the length and steps of the sliding window, these parameters were chosen directly by a function of the toolbox that optimizes both parameters: (1) the number of time points and (2) trials. P-optimum was evaluated using Akaike information criteria in the range of 5 to 20. The normalization applied to EEG raw data is called "ensemble normalization" and consists of point-wise subtracting the ensemble mean and dividing by the ensemble deviation.

Subsequently, all models were validated by checking their whiteness. A model is considered well estimated if the estimated noises U (equation S1) or residuals are small and uncorrelated. If the model was correctly estimated, its coefficients were used to calculate the gPDC connectivity values, else the model had evaluated again in a different order.

The computation of gPDC returns a matrix with a dimension 19×19 for each frequency, in this case, the frequency range was from 0.5 to 45 Hz with the step of 1 Hz. Single frequency matrices were then averaged in the same bands used in power spectra analysis (delta (0.5-4 Hz), theta (4-8 Hz), alpha (8-13 Hz), beta (13-30 Hz), gamma (30-45 Hz), total (0.5-45 Hz), and sigma (11-16 Hz)). Finally, for each subject, 18 weighted connectivity matrices were obtained (three epochs and six bands).

The definition of a threshold for obtaining binary matrices is still a discussed problem due to the high dependencies of the results on the threshold value. The choice of the

threshold also depends on the type of analysis that has to be performed. In this case, the bootstrap method was chosen and applied.

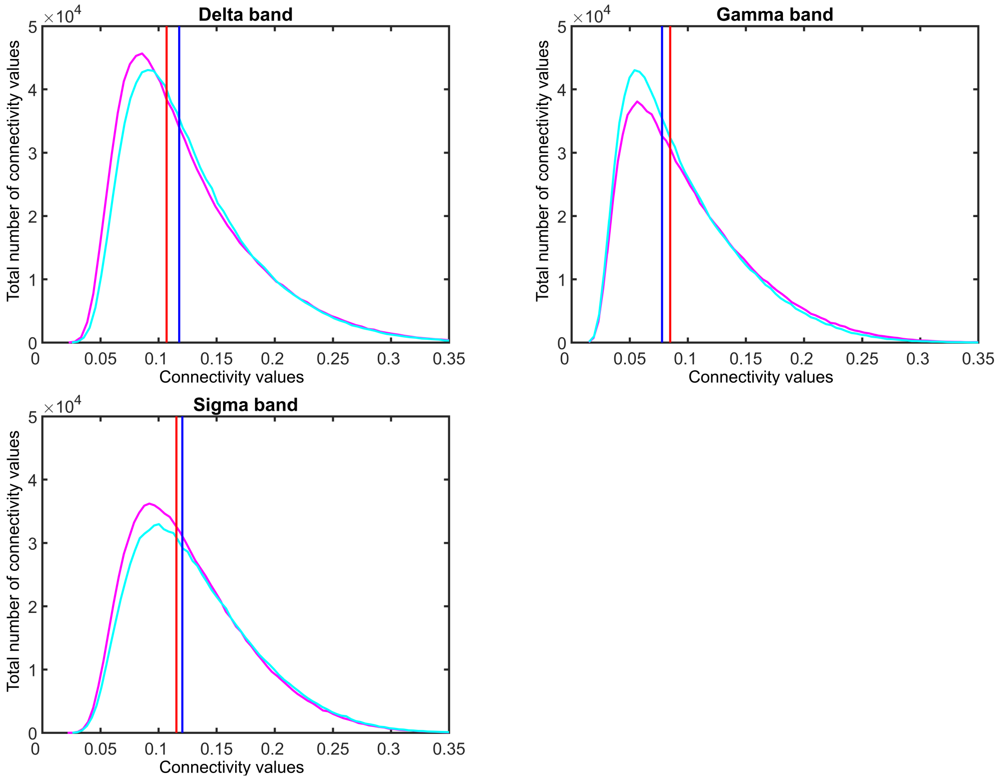

Figure S2. Representation of gPDC values distribution of delta, sigma, and gamma bands. The magenta line represents the distribution of ADHD connectivity values in all three epochs, while light blue represents the distribution of connectivity values of the Control in all the three epochs. Vertical lines represent the 50th percentile of the distribution, the red line is for ADHD distribution, blue is for Control.

This method is useful in cases of small sample size because it allows additional samples to be created, maintaining their fundamental characteristics. The creation of new signals was assessed by randomizing the phases of each channel of the EEG epochs. The randomization was repeated 100 times for each subject and epoch.
All bootstrapped signals were used to calculate the gPDC for all the seven bands delta, theta, alpha, beta, total, and sigma.

Each band was separately analyzed for each group by forming a distribution of the connectivity values obtained with the bootstrap method (Fig. S2). The threshold was chosen as the fiftieth percentile of the distribution in order to avoid a disconnected network, hence seven thresholds (one for each band) were calculated for each group.

4. Graph metrics

The binary connectivity matrices were obtained by applying the thresholds to each gPDC matrix of epochs and bands of original subjects. By means of the BCT Matlab toolbox, binary adjacency matrices were used to calculate, for each band and epoch, the in (Ki_{in}) and out (Ki_{out}) node degrees, cluster coefficient (Ci), and betweenness centrality (Bi) of each channel, and the mean cluster coefficient (C) and shortest path length (L) of the entire network.

Statistical differences were investigated between groups for all parameters, and among epochs only in C and L parameters. Differences were assessed via Wilcoxon sign rank test for paired samples, in the comparison of intra-group values; meanwhile, in between-groups

comparison, the Wilcoxon rank-sum test was applied. In addition, in the case of intra-group analysis, the Bonferroni correction was applied.

Finally, in order to evaluate the possibility of discriminating the two groups using these parameters, PCA was applied on (Ki_{in}) and (Ki_{out}) of all bands and epochs due to their characteristic results.
