# Supplementary figures and images for: Sleep Spindle-Related EEG Connectivity in Children with Attention-Deficit/Hyperactivity Disorder: An Exploratory Study
Source: Entropy (Basel). 2023 Aug 22;25(9):1244. doi: 10.3390/e25091244 (PMC10530036; doi:10.3390/e25091244)

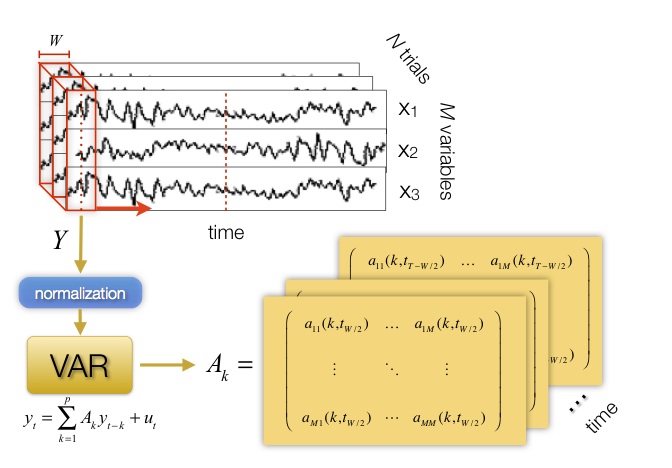

Supplement: Supplementary file 1 [file entropy-25-01244-s001.zip › C5/AMVAR.jpg]

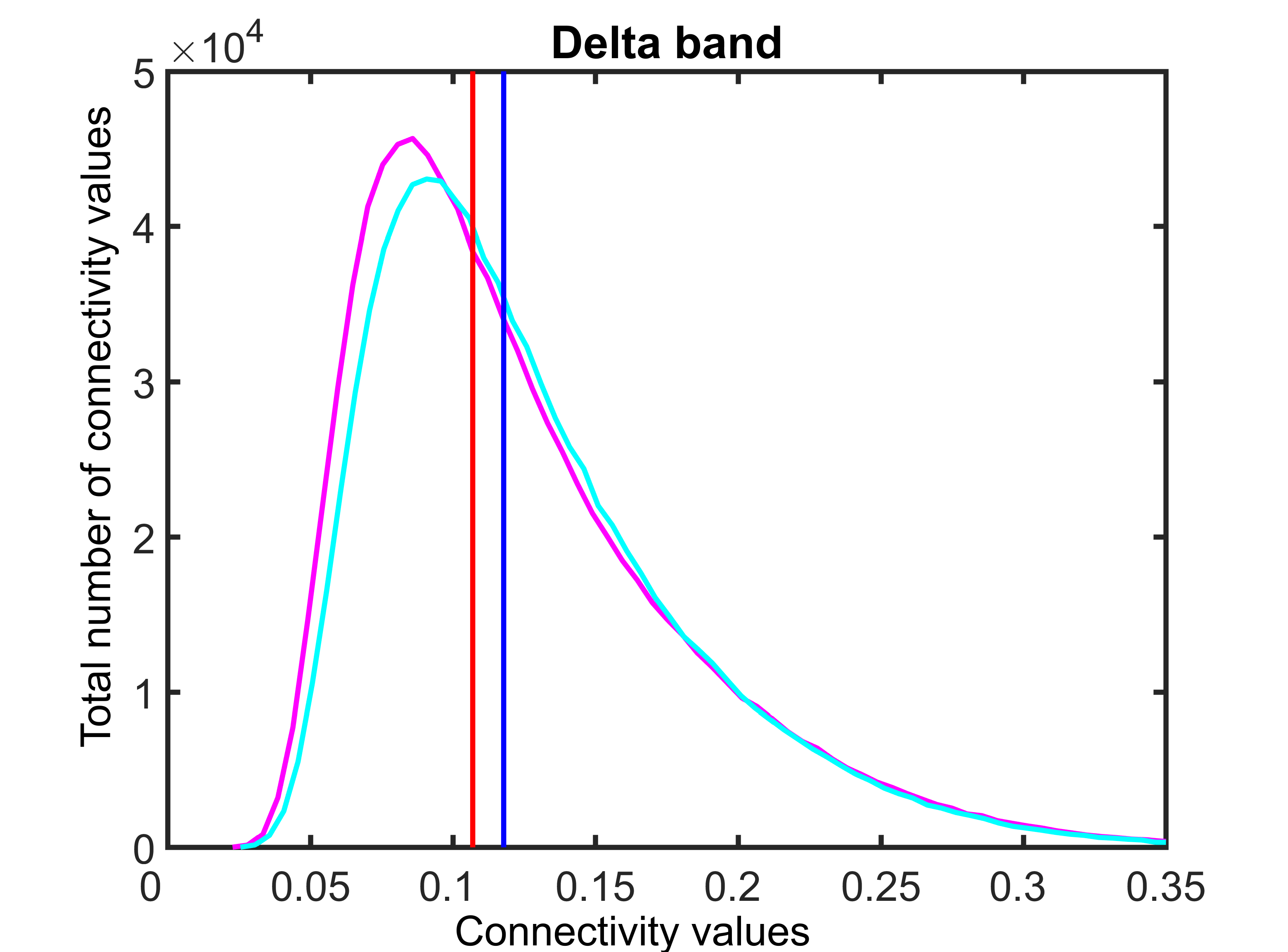

Supplement: Supplementary file 1 [file entropy-25-01244-s001.zip › C5/DeltaSoglia.png]

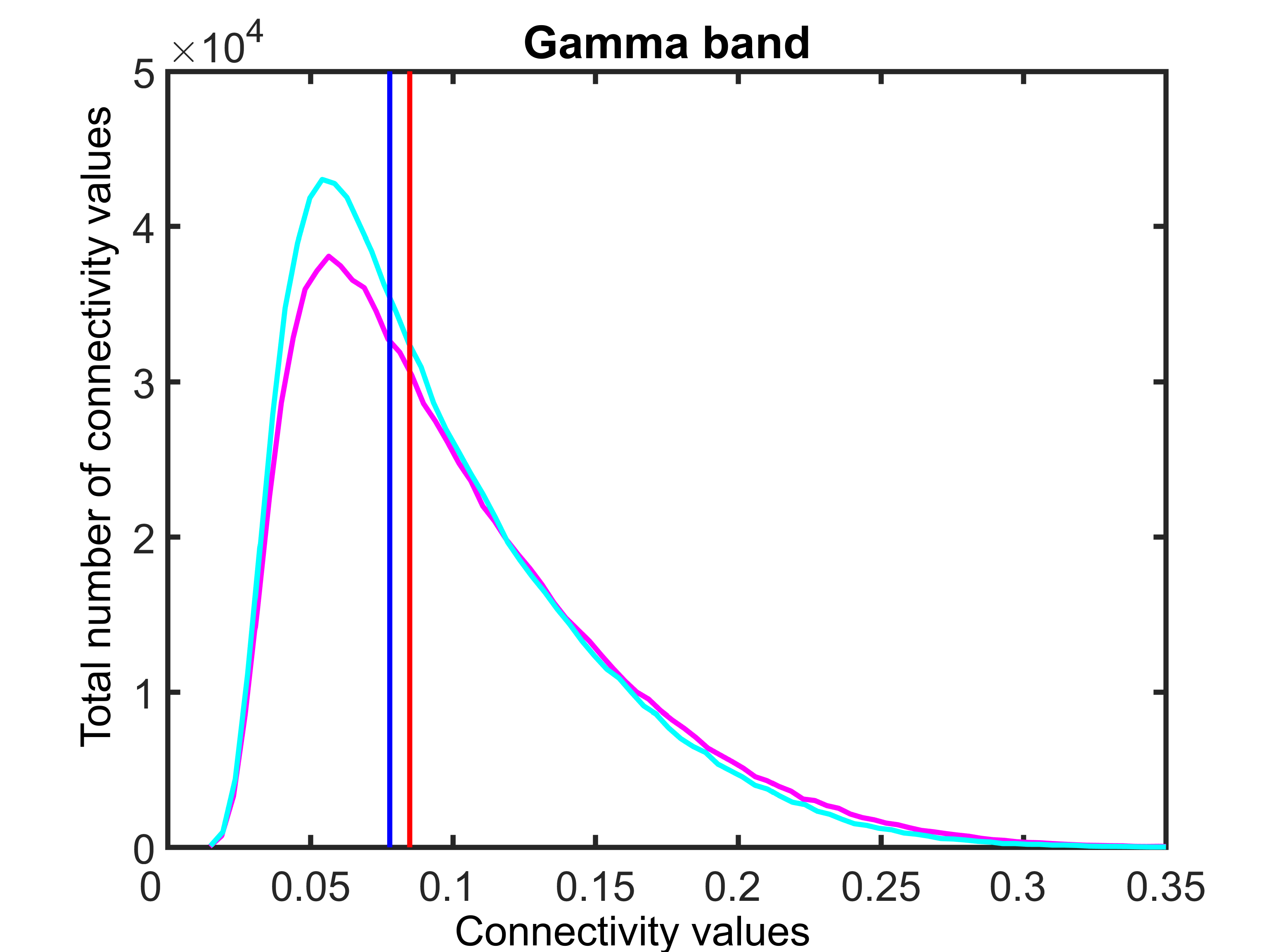

Supplement: Supplementary file 1 [file entropy-25-01244-s001.zip › C5/GammaSoglia.png]

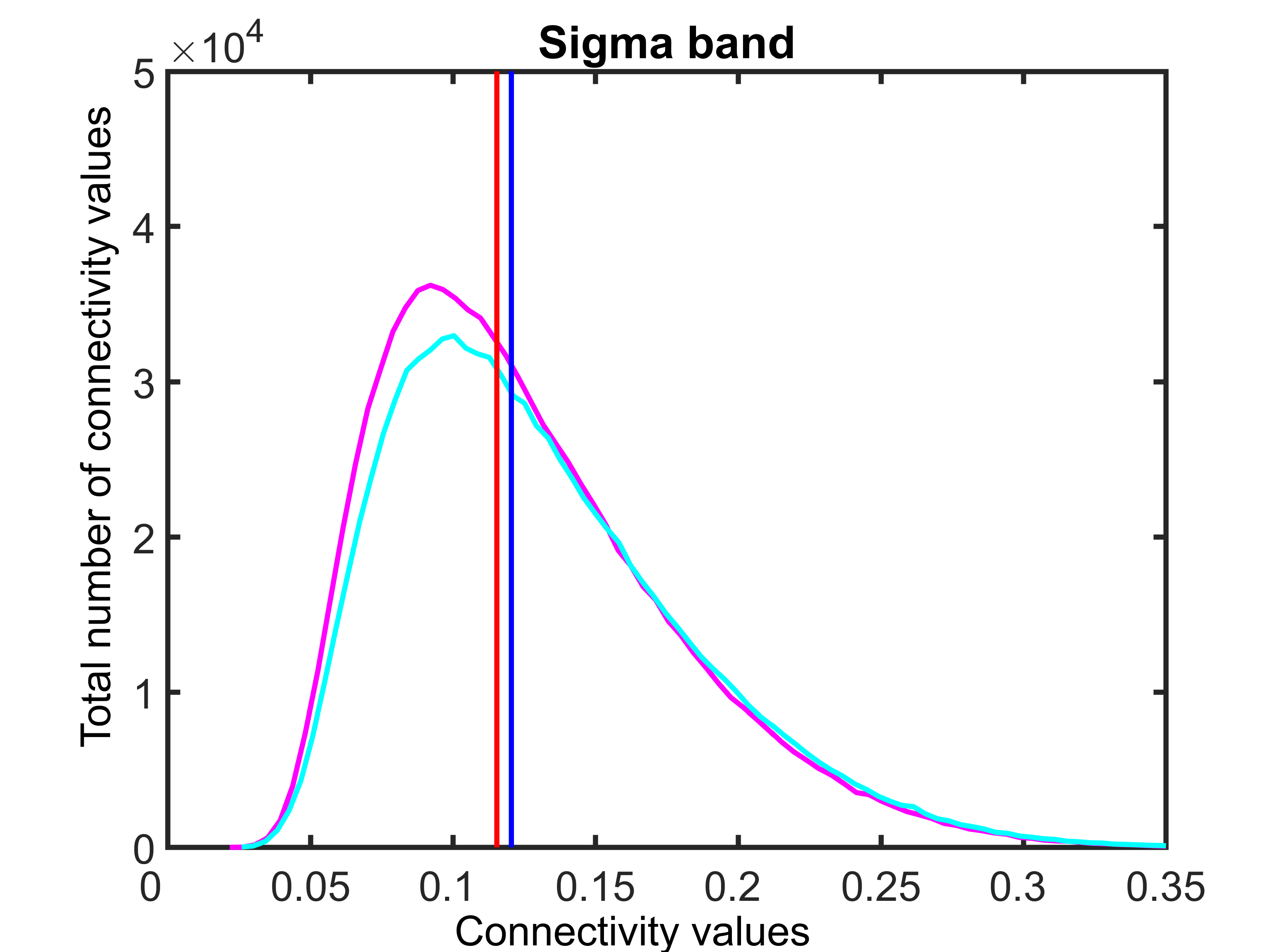

Supplement: Supplementary file 1 [file entropy-25-01244-s001.zip › C5/SigmaSoglia.png]

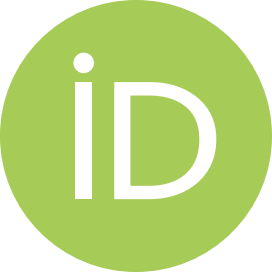

Supplement: Supplementary file 1 [file entropy-25-01244-s001.zip › Definitions/logo-orcid.pdf]

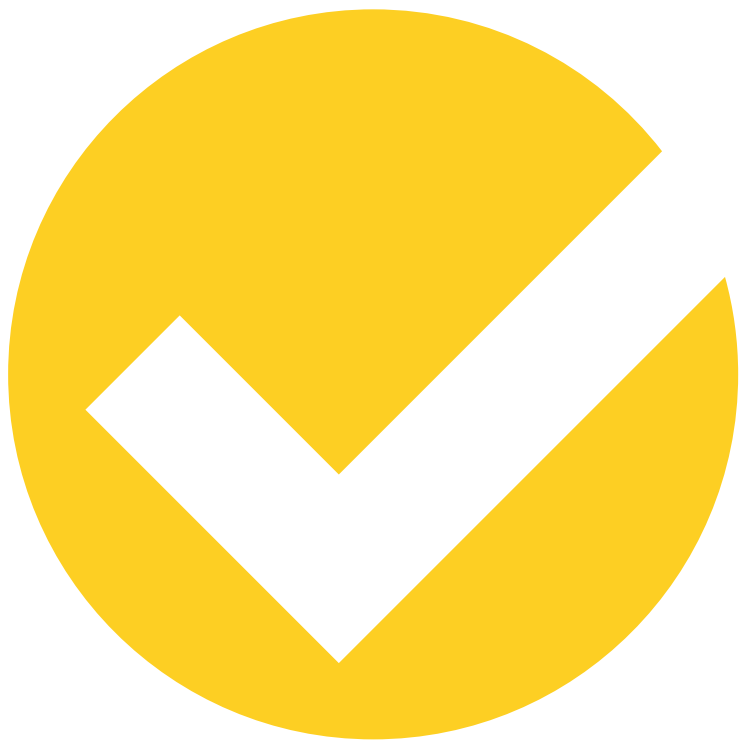

check for  
updates

Supplement: Supplementary file 1 [file entropy-25-01244-s001.zip › Definitions/logo-updates.pdf]
